# De-Esterified Homogalacturonan Enrichment of the Cell Wall Region Adjoining the Preprophase Cortical Cytoplasmic Zone in Some Protodermal Cell Types of Three Land Plants

**DOI:** 10.3390/ijms21010081

**Published:** 2019-12-20

**Authors:** Eleni Giannoutsou, Basil Galatis, Panagiotis Apostolakos

**Affiliations:** Section of Botany, Department of Biology, National and Kapodistrian University of Athens, 15781 Athens, Greece; egianno@biol.uoa.gr (E.G.); bgalatis@biol.uoa.gr (B.G.)

**Keywords:** preprophase band, de-esterified homogalacturonans, 2F4, JIM5, pectins, protodermal cells

## Abstract

The distribution of highly de-esterified homogalacturonans (HGs) in dividing protodermal cells of the monocotyledon *Zea mays*, the dicotyledon *Vigna sinensis*, and the fern *Asplenium nidus* was investigated in order to examine whether the cell wall region adjoining the preprophase band (PPB) is locally diversified. Application of immunofluorescence revealed that de-esterified HGs were accumulated selectively in the cell wall adjacent to the PPB in: (a) symmetrically dividing cells of stomatal rows of *Z. mays*, (b) the asymmetrically dividing protodermal cells of *Z. mays*, (c) the symmetrically dividing guard cell mother cells (GMCs) of *Z. mays* and *V. sinensis*, and (d) the symmetrically dividing protodermal cells of *A. nidus*. A common feature of the above cell types is that the cell division plane is defined by extrinsic cues. The presented data suggest that the PPB cortical zone-plasmalemma and the adjacent cell wall region function in a coordinated fashion in the determination/accomplishment of the cell division plane, behaving as a continuum. The de-esterified HGs, among other possible functions, might be involved in the perception and the transduction of the extrinsic cues determining cell division plane in the examined cells.

## 1. Introduction

The appearance of the cell wall during plant evolution triggered a wide range of adaptations in the plant cell function, completely deviating from the respective processes of animal cells. Among them, land plants have developed a unique interphase mechanism to define their division plane, a process not completely understood so far. This mechanism becomes evident by the organization of the preprophase band (PPB), a polarized cortical cytoplasmic zone, where the future cell plate fuses with the parent cell wall [1,2,3,4]. This is traversed by overlapping closed rings of microtubules (MT-PPB) [5], actin filaments (AF-PPB), [6] and, in some cases, tubular endoplasmic reticulum (ER-PPB) [7,8,9,10]. The ΕR-PPB is formed in cells in which the MTs consist of or contain acetylated tubulin [9].

Moreover, variable protein molecules are temporarily or permanently recruited in the cell division site (“positive markers of cell division”), whereas others are excluded from it (“negative markers of cell division”) [1,4]. Obviously, in the PPB cortical zone a mechanism functions, which either attracts or guides the cell plate at late cytokinesis to be anchored on the parent cell walls at predetermined sites [1,4,11,12,13].

In a definite cell type, the guard cell mother cell (GMC) of more than 20 Fabaceous species [14,15], as well as of *Arabidopsis thaliana* [16] the cell wall adjoining the PPB becomes locally thickened. The width of the MT-PPB coincides with that of the local cell wall thickenings, while the forthcoming cell plate bisects the latter with surprising accuracy [14,15,17]. Cell wall deposition at the PPB cortical site has been also assumed to happen in dividing root cells of *Allium cepa* [18] and moss protonemata [19], although cell wall thickening in the PPB cannot be clearly observed. Notably, in the Fabaceous species, the plasmalemma in the PPB region displayed numerous coated pits and many nearby coated vesicles [14,15]. Obviously, a preferential endocytotic route functions at the PPB region, a phenomenon later substantiated in living cells [20,21] as well as in cells following application of high pressure freezing and electron tomography techniques [22,23].

Preliminary data on GMCs and subsidiary cell mother cells (SMCs) of *Zea mays* revealed that the cell wall region lining the PPB was preferentially enriched with non-esterified homogalacturonans (HGs), which remained at this zone during cytokinesis. The forthcoming cell plate meets the parent cell walls at the regions premarked with the above pectin type [24].

The present article attempted to investigate whether the preferential cell wall matrix differentiation at the PPB cortical site represents a more general phenomenon of the protodermal cells of land plants. Accordingly, the distribution of highly de-esterified HGs, recognized by the JIM5 antibody [25], and non–esterified Ca^2+^-cross-linked HGs, recognized by 2F4 antibody [26], was examined in dividing protodermal cells of the monocotyledon *Zea mays*, the dicotyledon *Vigna sinensis*, and the fern *Asplenium nidus*. It has already been noted that the epitopes recognized by JIM5 and 2F4 antibodies are generally thought to increase cell wall stiffness [24]. Cells of the developing stomatal complexes were mainly studied because, in these, the division plane can be easily predicted by morphological criteria.

## 2. Results

**Figure 1:**
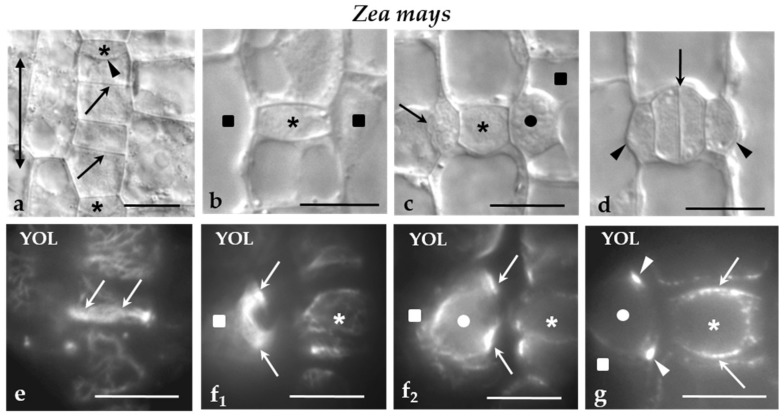
(**a**–**g**) *Z. mays* stomatal rows regions seen in (**a**‒**d**) DIC optics and (**e**‒**g**) after MT immunolabeling. Asterisks mark the GMCs, squares the SMCs and circles the nuclei. The double-headed arrow in (**a**) indicates the orientation of the leaf axis. (**a**) Part of a stomatal row near leaf meristem. The arrows point to the daughter cell walls of symmetrical divisions, while the arrowhead to the daughter cell wall of the asymmetrical division creating a GMC. (**b**) Newly-formed GMC. (**c**) GMC at the stage of induction of the adjacent SMCs becomes polarized and divides asymmetrically. The nucleus (circle) of the one SMC has been moved near the inducing GMC, while the other SMC has divided asymmetrically. The arrow points to the daughter cell wall of the asymmetrical division. (**d**) Newly-formed stomatal complex. The arrow shows the daughter cell wall of the symmetrical division of the GMC, while the arrowheads mark the daughter cell wall of the asymmetrical division separating the subsidiary cells. (**e**) Surface optical section of a preprophase cell of the stomatal row that is going to divide asymmetrically to create a GMC. The arrows indicate the MT-PPB. (**f_1,2_**) Optical sections passing through an (**f_1_**) external and (**f_2_**) a median plane of a preprophase SMC. The arrows point to the MT-PPB as seen in these two (**f_1_,_2_**) different planes. The circle marks the nucleus of the SMC. (**g**) Optical section through a median plane of a preprophase GMC. The arrows mark the MT-PPB. The arrowheads designate the MT-PPB in the adjacent SMC. The circle shows the nucleus of the SMC. Scale bars = 10 μm. Triangles are referred as arrowheads.

In *Z. mays*, the protodermal cells are arranged in rows aligned in parallel to the leaf axis. The cell divisions that occurred in them are: (a) symmetrical divisions that multiply the protodermal cells (Figure 1a), (b) asymmetrical divisions of the stomatal row cells that produce the GMCs (Figure 1a,b), (c) asymmetrical divisions of the SMCs, which generate the subsidiary cells of the stomatal complexes (Figure 1c,d), and (d) symmetrical division of the GMCs, which gives rise to the guard cell pair (Figure 1d). In the first two types of cell divisions, the plane of cell division is aligned transversely to the leaf axis (Figure 1a,b), while in the rest it is parallel to leaf axis (Figure 1c,d). In all of them, a well-organized MT-PPB accurately predicts accurately the sites of junction of the future cell plate with parent cell walls (Figure 1e–g).

**Figure 2:**
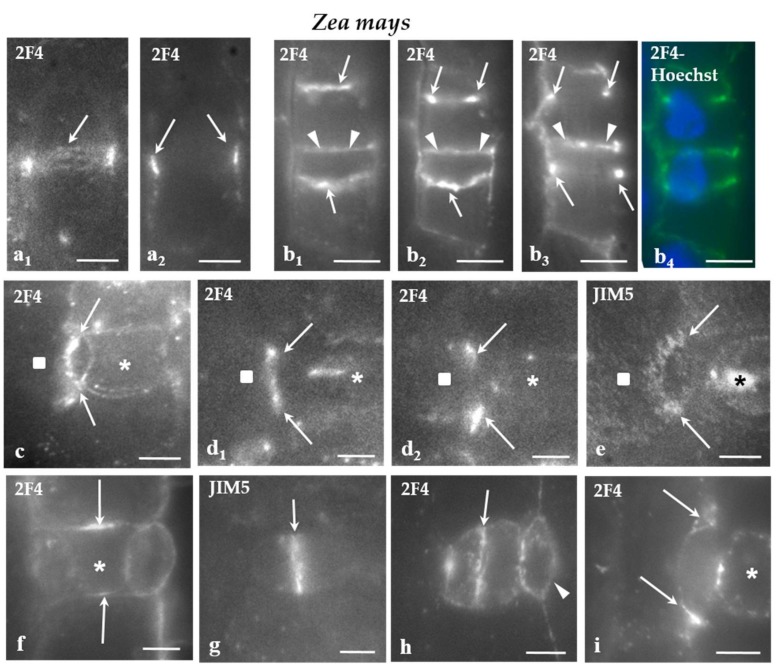
(**a**–**i**) Immunolabeling of JIM-5 and 2F4-HG epitopes in stomatal row cells of *Z. mays.* Asterisks mark the GMCs and squares the SMCs. (**a_1,2_**) Paradermal optical sections passing through (**a_1_**) a surface and (**a_2_**) a median plane of a stomatal row cell that is going to divide symmetrically. The cell wall region adjoining the PPB region emitted a local 2F4 fluorescent signal (arrows). (**b_1_–_4_**) Paradermal (**b_1,2_**) surface and (**b_3_**) median optical sections of two cells of the stomatal row that were programmed divide asymmetrically. The cell wall region adjacent to the PPB (arrows) emitted a distinct 2F4 fluorescent signal (compare to Figure 1e). The arrowheads points to the daughter cell wall of the symmetrical division of a stomatal row cell that also emits 2F4 fluorescent signal. (**b_4_**) The nuclei of the cells that will divide asymmetrically as seen after DNA staining with Hoechst 33258. (**c**) Surface paradermal optical view of an SMC. The cell wall at the PPB region (arrows) emitted a local fluorescent 2F4 signal (compare to Figure 1 f_1_). (**d_1_,_2_**) Paradermal optical sections through (**d_1_**) a surface and (**d_2_**) a median plane of an SMC. The cell wall at the PPB region (arrows) emitted a local 2F4 fluorescent signal (compare to Figure 1 f_1,2_,g). (**e**) Surface paradermal optical section of an SMC. The cell wall outlining the PPB region (arrows) locally emitted a JIM5 fluorescent signal (compare to Figure 1f). (**f**) Median paradermal optical section of a GMC. The cell wall region external to the PPB (arrows) emitted 2F4 fluorescent signal (compare to Figure 1g). (**g,h**) Median paradermal optical sections of newly formed stomatal complexes. The daughter cell wall (arrows) of the GMC symmetrical division displayed an intense (**g**) JIM5 and (**h**) 2F4 fluorescent signal. The arrowhead in (**h**) marks the daughter cell wall of the asymmetrical SMC division, which emitted a weak 2F4 fluorescent signal. (**i**) Paradermal optical section through a median plane of a divided SMC. The margins of the daughter cell wall of the asymmetrical division of the SMC emitted 2F4 fluorescent signal (arrows). Scale bars = 5 μm.

In *Z. mays*, the cell wall adjoining the PPB cortical zone in protodermal cells dividing symmetrically (Figure 2a_1,2_), those dividing asymmetrically to produce the GMCs (Figure 2b_1_–_4_), SMCs (Figure 2c–e), as well as symmetrically dividing GMCs (Figure 2f) was preferentially enriched by the 2F4- and JIM5-HG epitopes. The epitope accumulation was more intense in the cells dividing asymmetrically (Figure 2b–e). In SMCs, the JIM5 and 2F4 fluorescent cell wall region was located externally to the peculiar-in-shape PPB cortical zone (Figure 2c,d_1_,e; cf Figure 1f_1_ and Figure 2d_2_; cf. Figure 1f_2_,g). In all of these divisions, the daughter cell wall emitted JIM5 and 2F4 fluorescent signals (Figure 2g–i) that were more intense at their periphery.

**Figure 3:**
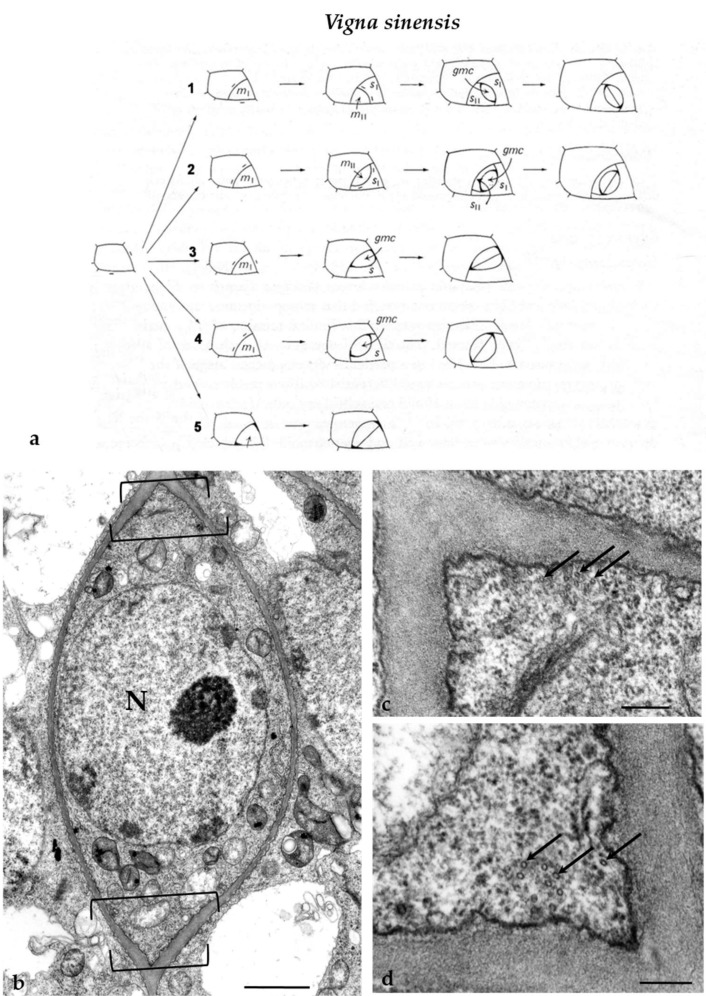
(**a**) Diagrammatic representation of the successive developmental stages of *V. sinensis* stomatal complexes. In (**a_1_,_2_**) the development of the stomatal complexes was accomplished by three asymmetrical cell divisions, creating a lens-shaped GMC and two subsidiary cells, and one symmetrical division of the GMC giving rise to the guard cells. In (**a_3_,_4_**), the development of the stomatal complexes included two asymmetrical divisions giving rise to a (**a_3_**) triangular or (**a_4_**) a lens-shaped GMC and a subsidiary cell and was completed with the symmetrical division of the GMC. In (**a_5_**), a stoma lacking subsidiary cells is depicted. Its development was carried out by one asymmetrical division, generating the GMC, and a symmetrical division of the last, resulting in the two guard cells. The bars define the location of the PPB in the asymmetrically dividing cells as it appeared in paradermal sections. The position of the PPB in GMCs was characterized by deposition of local cell wall thickenings. GMC: guard cell mother cell; (m_I_, m_II_): meristemoids; (s, s_I_, s_II_): subsidiary cells (modified by Galatis and Mitrakos [14]). (**b**) Median paradermal section of a preprophase GMC of *V. sinensis* as seen in TEM. The brackets delimit the site of the MT-PPB. Note the local cell wall thickening deposited externally to the PPB site. N: nucleus. Scale bar = 1μm. (**c**,**d**) PPB sites of the GMC in (b) at higher magnification. The arrows point to MTs of the PPB. Scale bars = 200 nm.

The ordinary protodermal cells of *V. sinensis* divided symmetrically in a plane usually aligned perpendicularly to the long cell axis as well as asymmetrically, initiating the process of stomatal complexes development. The latter was usually accomplished by one to three asymmetrical divisions and a symmetrical one that formed the guard cells (Figure 3a). The number and the plane of asymmetrical divisions involved in stomatal complexes development, which leads to the formation of either lens-like or triangular GMCs, are shown in Figure 3a. Regardless of their shape, the GMCs display a well-organized MT-PPB [14] (see also Figure 3b–d). The cell wall adjacent to the PPB cortical zone is thickened [14]. Well organized MT-PPBs foreshadow the sites of fusion of the daughter cell wall of symmetrical divisions of the protodermal cells as well of the asymmetrical ones involved in stomatal complexes development [14]. The plane of the symmetrical GMC division was parallel to that of the preceding asymmetrical division (Figure 3a).

**Figure 4:**
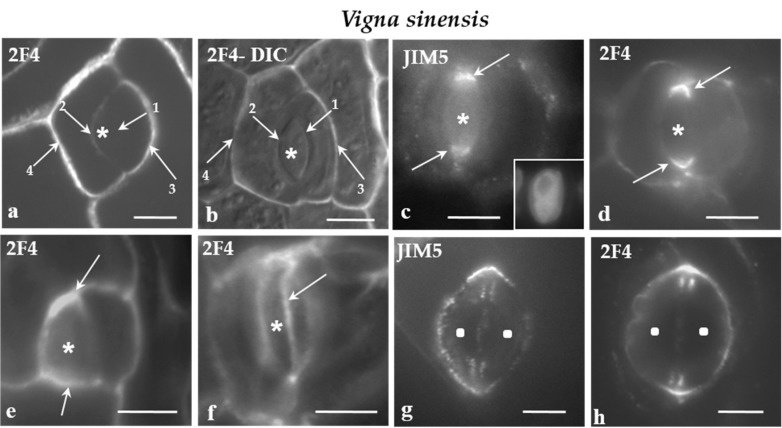
**a-h**: Immunolabeling of JIM5- and 2F4-HG epitopes in developing stomatal complexes of *V. sinensis*. Asterisks mark the GMCs and squares the guard cells. (**a,b**) Immunolabeling of the 2F4-HG epitope in stomatal complexes at the stage of GMC (asterisks). (b) The stomatal complex as seen in a merged photo of DIC optics and 2F4 immunolabeling. The arrows 1–4 show cell walls from the youngest (arrow 1) to the most mature (arrow 4; Compare to Figure 3a). It was obvious that the intensity of the fluorescent signal is proportional to the maturation stage of the cell wall. (**c,d**) Lens-shaped GMCs as seen in median paradermal optical sections after immunolabeling of JIM5- and 2F4-HG epitopes. The cell wall thickenings deposited externally to the PPB (see Figure 3b–d) emitted intense fluorescent signal (arrows). Inset: The nucleus of the GMC as seen after DNA staining with Hoechst 33258. (**e**) Median paradermal optical section of a triangular GMC after immunolabeling with the 2F4 antibody. An intense fluorescent signal (arrows) emanated from the cell wall region adjoining the PPB (compare to Figure 3a_3_). (**f**) Surface optical section of a lens-shaped GMC. The arrow points to a narrow zone of the periclinal cell wall displaying a localized and intense 2F4 fluorescent signal. This fluorescent zone of the periclinal cell wall corresponds to cell wall region adjacent to the periclinal part of the PPB. (**g,h**) Immunolabeling of the JIM5- and 2F4-HG epitopes in young stomatal complexes. The margins of the daughter cell wall of the GMC symmetrical division (see Figure 3a) emitted a distinct local (**g**) JIM5 and (**h**) 2F4 fluorescent signal. Scale bars = 5 μm.

All of the cell walls of the protodermal cells of *V. sinensis* were enriched with the JIM5- and the 2F4-HG epitopes. Moreover, in parallel to the cell walls differentiation, the participation of JIM5- and 2F4-HG epitopes in their composition increased. In developing stomatal complexes, where the sequence of cell wall formation can be easily traced, the intensity of the fluorescent signal increased from the younger cell walls to the older ones (Figure 4a,b).

In *V. sinensis*, the JIM5 and 2F4-HG epitopes were preferentially accumulated along the whole anticlinal cell wall thickenings externally lining the PPB region in both lens-shaped and triangular GMCs (Figure 4c–e), as well as along the cell wall sites adjacent to the periclinal PPB regions (Figure 4f). The same epitopes were also traced in the daughter cell wall of the GMC division. Their presence was more intense at the periphery of the daughter cell wall (Figure 4g,h).

**Figure 5:**
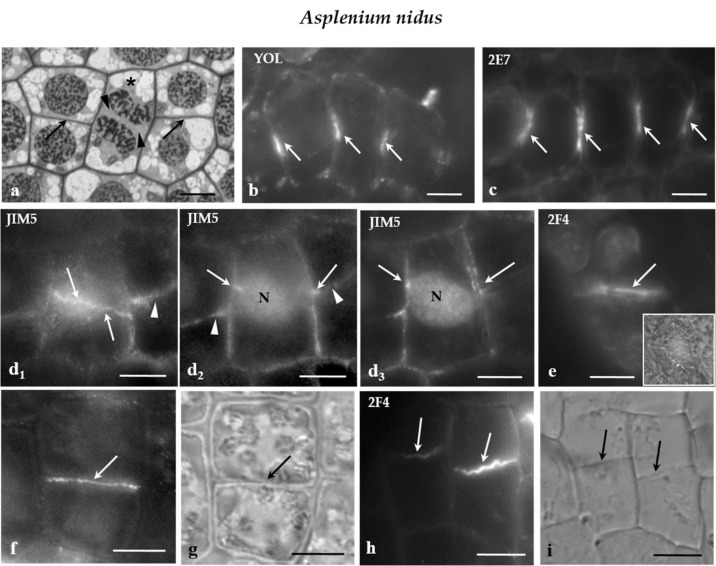
(**a**) Median paradermal section of a group of *A. nidus* protodermal cells, as seen in light microscope after toluidine blue staining. The asterisk marks a cell in cytokinesis. The arrows point to daughter cell walls of symmetrical divisions that are arranged on the same plane. The arrowheads show the cell plate of the cytokinetic cell. (**b**,**c**) Median optical sections of groups of *A. nidus* protodermal cells, where (**b**) MT and (**c**) ER membrane immunolabeling has taken place. The arrows point to profiles of (**b**) MT-PPBs and (**c**) ER-PPBs. (**d_1_**) Surface and (**d_2,3_**) median optical sections of an *A. nidus* protodermal cell, as seen after immunolabeling with the JIM5 antibody. The cell wall in the PPB region (arrows) emitted intense fluorescent signal. The arrowheads point to the daughter cell wall at the neighboring cells. Note that the PPB and the daughter cell wall were located on the same plane. (**e**) Surface optical section of an *A. nidus* protodermal cell, as seen after immunolabeling with 2F4 antibody. The arrow indicates the cell wall at the PPB site that emitted a local fluorescent signal. The inset in (**e**) illustrates the protodermal cell, as seen in DIC optics. (**f**,**g**) Symmetrically divided *A. nidus* protodermal cell, as it appeared after immunolabeling with (f) JIM5 antibody and (g) DIC optics. The daughter cell wall (arrow in g) emitted intense fluorescent signal (arrow in f). (**h**,**i**) Symmetrically divided *A. nidus* protodermal cells, as they appeared after immunolabeling with the (**h**) 2F4 antibody and (**i**) DIC optics. The daughter cell walls (arrows in i) emitted 2F4 fluorescent signal (arrows in h). Scale bars = 5 μm.

The protodermal leaf cells of the fern *A. nidus* divided symmetrically to proliferate the ordinary protodermal cells and asymmetrically to initiate stomatal complexes [27]. In the protoderm of this species, certain groups the daughter cell walls were laid down on the same plane (Figure 5a), a phenomenon showing that the cell division plane is strictly coordinated between neighboring cells. Since these cells encompass well-sized vacuoles, the cell division plane was usually evident during interphase by organization of a distinct phragmosome, at the periphery of which the MT-PPB and ER-PPB were organized [10] (See also Figure 5b,c). Frequently, the phragmosomes between adjacent cells were organized on the same level [10] (see also Figure 5b,c).

Examination of the immunofluorescent specimens revealed that the JIM5- and 2F4-HG epitopes were preferentially accumulated in the cell walls external to the PPB region only in the symmetrical protodermal cell divisions (Figure 5d_1_–e) as well as in their daughter cell walls (Figure 5f–i). In cells of the developing stomatal complexes, for example the GMCs, and the JIM5- and 2F4-HG epitopes were dispersed along the whole surface of the cell walls, without any preferential localization to the PPB cortical zone. It should be noted that the plane of the symmetrical GMC division was perpendicular to the previous one that generated the GMC [27]. The above data support the view that, although it is far from being a phenomenon observed in all dividing cell types, the cell wall region adjoining the PPB cortical zones of some of the symmetrically and asymmetrically dividing protodermal cells of the examined plants was enriched with the JIM5- and 2F4-HG epitopes.

## 3. Discussion

The division plane in plants is defined by internal or external cues depending on the cell type. In the first case, the geometry of the cell seems to play the primary role. According to [28] and [29], the daughter cell wall is laid down transversely to the long cell axis, tending to assume the minimal possible surface area. Although this rule can be applied for many cell types, there are many exceptions [2,4,30,31,32]. In the second case, mechanical stresses [33,34], auxin signaling [35,36,37], and other polarizing cues (reviewed by [3,4,38]) play the decisive role in the determination of the cell division plane.

The data presented in this work show that in the (a) symmetrically dividing cells of the protodermal rows of *Z. mays*, (b) asymmetrically dividing protodermal cells forming the GMCs in *Z. mays*, (c) SMCs of *Z. mays*, (d) GMCs of *Z. mays* and *V. sinensis*, and (e) symmetrically dividing protodermal cells of *A. nidus*, the cell wall on the cell division plane becomes locally differentiated by the local accumulation of highly de-esterified HGs in the cell wall adjoining the PPB cortical zone. Since HG demethylesterification is mediated by pectin methylesterases [39], it can be assumed that a local activation of these enzymes occurs in the cell wall outlining the PPB cortical zone. Consideration of the existing information and the present findings further support the views that: (a) the PPB cortical zone is a very complicated and active cytoplasmic region, where a series of unique cytoskeletal and membranous structures are organized and (b) PPB cortical zone-plasmalemma and the adjacent cell wall function in a coordinated fashion during the determination/accomplishment of the cell division plane. Regardless of the particular role of each of them in cell division plane definition, the present data support the hypothesis that these zones function as a continuum during the above process. In particular, although, regarding the role of the local differentiation of the cell wall at the PPB cortical zone, only hypotheses can be made, it is reasonable to suggest that it is involved in the determination of the cell division plane.

Since the de-esterification of the HGs at the PPB cortical zone appeared only in certain types of protodermal cells, to understand the phenomenon we have considered their common characteristic(s) related to the cell division plane determination. In the cell types in which the phenomenon appeared, the premitotic polarization and the determination of the cell division plane seem to be controlled by external cues.

In leaf protoderm of graminaceous species, the polarization of the stomatal row cells that divide asymmetrically to produce the GMCs seems to be a hormone-dependent process [40]. Moreover, in *Z. mays*, auxin is probably the inducing stimulus that is emitted by the GMCs and triggers the polarization/asymmetrical SMC division [41,42]. Numerous molecules are likely involved in the transduction of this stimulus [43] in which de-esterified HGs are included [24,43].

In protodermal cells of *A. nidus*, the cell division plane is predicted during interphase with phragmosome formation at the periphery of which the MT-PPB and the ER-PPB are assembled [10]. In this plant, the plasmalemma and the nuclear envelope also displayed local polarization on the phragmosome plane. Interestingly, the phragmosome, the MT-PPB (Figure 5b), and the ER-PPB (Figure 5c) organization, as well as the plasmalemma and the nuclear membrane differentiation in the PPB region between neighboring cells, often occur on the same plane [10]. Thus, in large protodermal areas of *A. nidus*, an induction stimulus related to intercellular induction signal(s) seems to spatially coordinate the alignment of the division plane between adjacent cells [10]. Experimental data of the same authors suggest that the induction signal(s) is emitted by the apical region of the *A. nidus* leaf. Considering that the growing leaf tips function as auxin source sites [44], the induction signals that determine the cell division plane in the protoderm of *A. nidus* are probably hormonal in nature [10].

In dividing SMCs of *Z. mays*, the daughter cell wall is laid down in parallel to the long cell axis and, furthermore, is oriented parallel to the leaf axis (Figure 1c,d). The SMCs are one of the few dividing cell types of the *Z. mays* meristem, where the daughter cell wall is laid down in parallel to the leaf axis (Figure 1c,d; cf. Figure 1a). There are data suggesting that, in cells dividing in parallel to the long cell axis, the cell division plane is defined by mechanical stresses exerted on the dividing cell (reviewed by [4,30]). It has been suggested that in SMCs of *Z. mays*, the cell division plane is controlled by local mechanical stresses applied on them by the laterally adjacent GMCs [40,45,46]. Moreover, the highly de-esterified HGs that accumulate on the cell wall at the polar end of the SMCs are possibly involved in the generation of these mechanical stresses [24,43]. It is interesting that, in SMCs, the division plane is defined even when the cell division has been experimentally inhibited [41,42,46]. These data further enforce the view that mechanical forces play a critical role in the determination of the plane of plant cell division.

The dividing GMC represent the second *Z. mays* protodermal cell type where the daughter cell wall is laid down in parallel to the leaf axis (Figure 1d). Mechanical stresses possibly determined the cell division plane in GMCs. Before their division, the GMCs followed an accurately determined pattern of morphogenesis, expressed by definite changes in their dimensions (Figure 1c; cf. Figure 1b). Their length, i.e., the dimension that is parallel to the stomatal row axis doubles (from 4.27 ± 0.1 to 9.62 ± 0.1μm), while their width, i.e., the dimension that is perpendicular to the stomatal row axis, reduces from 13.46 ± 0.1 to 8.61 ± 0.1μm [47]. At the same time, the SMCs grow towards the GMCs (review by [43]), while the proximal and distal to GMCs intervening cells of the stomata rows grow significantly in width [47]. Therefore, the surrounding cells should exert mechanical stress on GMCs. Besides, the GMCs appear constricted at their middle since a mechanism preventing their growth in width functions in them. Early in their development, a cellulose microfibril band is deposited at the middle of their lateral anticlinal and periclinal cell walls, externally to an MT-band, which favors their elongation but prevents their widening [40,48]. This constriction appears to be an early step of dumbbell-shape guard cell morphogenesis, which seems to start at the GMC stage [40,48,49]. During this process, additional mechanical stresses are exerted on particular sites of GMCs. Therefore, the division plane of *Z. mays* GMCs seems to be defined by the interaction of mechanical forces exerted by the surrounding cells with those generated in the GMC itself.

In dividing GMCs of *V. sinensis*, the daughter cell wall is oriented in parallel to the long cell axis (Figure 3a). The mechanism controlling the kidney-shape guard cell morphogenesis in *V. sinensis*, in which the tangential expansion of the dorsal anticlinal cell wall plays the main role, seems to be activated at the GMC stage [14,40,50]. As a result, mechanical forces are exerted at the junction of the anticlinal cell walls. At these positions the PPB appears and local cell wall thickenings are deposited, which are inherited by the guard cells [17]. These thickenings possibly counterbalance the forces exerted on these positions during the opening of the stomatal pore [51]. Thus, mechanical forces may be involved in the definition of the plane of cell division in the GMCs of *V. sinensis.*

Regarding the involvement of the JIM5- and 2F4- HG epitopes in determining the plane of cell division of the examined cells, only hypotheses can be made. It is known that: (a) oligogalacturonide and other oligosaccharin molecules, derived from the breakdown of HGs and other materials of the cell wall matrix, function as signal transduction molecules and (b) oligosaccharides are generated in cell wall regions, where highly de-esterified HGs dominate (reviewed by [39,52]). Therefore, the JIM5- and 2F4- HG epitopes enriching the cell wall at the PPB region may participate in the transduction of hormonal stimuli involved in the determination of the cell division plane between neighboring cells, as in the case of *A. nidus*. It has already been suggested that HGs participate in the transduction of the auxin stimulus in the protoderm of *Z. mays* [24,43].

When mechanical forces define the plane of cell division, this is probably achieved via MTs that sense mechanical stresses and respond to them [53,54]. As a result, the MT-PPB is assembled parallel to the tensile stress maxima defined by the mechanical forces exerted on the dividing cells [33,34] (reviewed by [4,32]). Although the process by which mechanical stress is perceived by the cell and the cytoskeleton is not completely understood, a mechanosensing mechanism for the perception and the transduction of the mechanical stresses may function in the cells. Molecules or some pectin fragments seem to participate in this mechanism [4]. Considering that pectin fragments are generated in cell wall regions where de-esterified and non-esterified HGs dominate [39], the JIM5- and 2F4-HG epitopes localized at the PPB region might contribute to the establishment of the mechanosensing mechanism functioning at this region.

It has already been supported that plasmalemma-derived endocytic vesicles transfer parental cell wall material, such as de-esterified HGs, to the cell plate, via endosomes [21,55,56]. The experimental disturbance of the fusion of endocytic vesicles with endosomes results in the absence of de-esterified HGs at the cell plate [57]. Taking into account that (a) intense endocytosis takes place at the PPB region [20,21,22,23] and (b) the daughter cell walls of the dividing cells studied in this work were rich in JIM5- and 2F4-HG epitopes (Figure 2g–I, Figure 4g,h and Figure 5f,h), it can be assumed the premitotic accumulation of JIM5- and 2F4-HG epitopes at the PPB region can function as a source of de-esterified HGs towards the cell plate during cytokinesis. However, this view requires further thorough investigation.

In conclusion, it may be suggested that when the plane of cell division is determined by extrinsic factors, not only the cytoskeleton, the ER membranes and the plasmalemma, but also the cell wall adjoining to the PPB cortical zone, seem to be involved in the definition of the cell division plane, functioning as a united system with the former PPB cytoplasmic elements.

## 4. Materials and Methods

### 4.1. Plant Material

This study was carried out in young leaves of *Zea mays* L. Var Aris, *Vigna sinensis* L., and *Asplenium nidus* L. To examine early stages of protodermal development of *Z. mays*, *Z. mays* seedlings were grown in small beakers on filter paper soaked with distilled water for 3–5 days in darkness, at 25 ± 1 °C. *Z. mays* caryopses were kindly offered by the National Agricultural Research Foundation, Cereal Institute (Thessaloniki, Greece). *V. sinensis* seedlings were grown in beakers filled with perlite under room conditions for 5–15 days. *A. nidus* plants were grown in a greenhouse and were further developed in the laboratory. For the specific study, the apical part of *A. nidus* young leaves was used.

### 4.2. Microtubule (MT) and Endoplasmic Reticulum (ER) Immunolocalization

For MT and ER immunolocalization, the protocol described by Giannoutsou et al. [10] was applied. In detail, paradermal hand-made leaf sections were fixed in 1% (*w*/*v*) paraformaldehyde (PFA) in PEM buffer (50 mM PIPES, 5 mM EGTA, 5 mM MgSO_4_, pH 6.8), for 20 min at room temperature and then in 4% (*w*/*v*) PFA in the same buffer for another 20 min. After a thorough washing with PEM, the material underwent a mild cell wall digestion with 1% (*w*/*v*) pectinase (Sigma, St.Louis, MO, USA), 1% (*w/v*) cellulase (Yakult Honsha, Tokyo, Japan), 2% (*w/v*) driselase (Sigma, St.Louis, MO, USA), and 1% (*v/v*) glucuronidase (Sigma, St.Louis, MO, USA) in PEM, pH 5.6, for 20 min. Following rinsing with PEM, the material was treated for 20 min with 0.5% (*v/v*) Triton X-100 and 2% (*v/v*) DMSO in phosphate buffered saline (PBS). Then, the samples were washed with PBS containing 1% (*w/v*) bovine serum albumin (BSA), followed by an overnight incubation with the appropriate primary antibody diluted 1:40 in PBS containing 1% (*w/v*) BSA at room temperature. After rinsing with PBS, containing 1% (*w/v*) BSA, the samples were incubated with the appropriate secondary antibody diluted 1:40 in PBS containing 1% (*w/v*) BSA, for 2 h at 37 °C. After washing with PBS, the DNA was stained for 5 min with 10 μg ml^−1^ Hoechst 33258 Sigma, St.Louis, MO, USA) in PBS and the samples were mounted with an anti-fade solution [2.4 mg p-phenylenediamine (Sigma, St.Louis, MO, USA) diluted in 1.5 mL of a solution containing 2:1 glycerol:PBS].

For the MT immunolocalization, a rat monoclonal anti-α-tubulin antibody clone YOL 1/34 (Serotec, Oxford, UK) was used as primary antibody and a fluorescein isothiocyanate (FITC)-conjugated anti-rat IgG (Sigma, St.Louis, MO, USA), as a secondary antibody. For the ER immunolocalization, 2E7 (HDEL (2E7): sc-53472, Santa Cruz Biotechnology Inc, Texas, USA) as a primary antibody was used and FITC-conjugated anti-mouse IgG (Sigma, St.Louis, MO, USA) was used as a secondary antibody.

### 4.3. Immunolocalization of Homogalacturonans

For immunolabeling of JIM5- and 2F4-HG epitopes in fixed freehand leaf sections, the protocol described by Giannoutsou et al. [24] was applied. JIM5 and 2F4 (Plant Probes, Leeds, UK) were used as primary antibodies and FITC–conjugated anti-rat and anti-mouse IgG (Sigma) were used as secondary antibodies, respectively. JIM5 antibody was diluted 1:40 in PBS that contained 2% (*w/v*) BSA. 2F4 and its secondary antibody were diluted 1:40 in T/Ca/S buffer (Tris-HCl 20 mM pH 8.2, CaCl_2_ 0.5 mM, NaCl 150 mM). During the immunolabeling procedure with 2F4 antibody, the sections were washed with T/Ca/S buffer (for details see plant probes leaflet).

### 4.4. Transmission Electron Microscopy and Light Microscopy

Small pieces of leaves were fixed in glutaraldehyde, post-fixed in osmium tetroxide, dehydrated in an acetone series, and embedded in either Durcupan ACM (Fluka, Munich, Germany) or Spurr’s mixture (Serva, Heidelberg, Germany). Thin sections were stained with uranyl acetate and lead citrate. Semithin sections were stained with 0.5% (*w/v*) toluidine blue in 1% (*w/v*) borax solution.

### 4.5. Observation and Photography

Semithin and hand-made sections were examined with a Zeiss Axioplan microscope (Zeiss, Berlin, Germany) equipped with a UV source, a differential interference contrast (DIC) optical system, and the proper filters: a filter set provided with exciter solid glass filter 365 nm and barrier longwave pass band filter 420 nm and another set provided with exciter pass band filter 450–490 nm and barrier pass band filter 515–565 nm, while all the photos were taken using a Zeiss Axiocam MRC5 digital camera (Zeiss, Berlin, Germany). Thin sections were examined with a Philips 300 Transmission Electron Microscope (TEM, Philips, Eindhoven, the Netherlands).

## Abbreviations

PPB	Preprophase band
MT-PPB	Microtubule-preprophase band
ER-PPB	Endoplasmic reticulum-preprophase band
AF-PPB	Actin filament preprophase band
GMC	Guard cell mother cell
SMC	Subsidiary cell mother cell
HG	Homogalacturonan
